# Crystal structure of nafamostat dimesylate

**DOI:** 10.1107/S2056989021009245

**Published:** 2021-09-10

**Authors:** Isao Fujii

**Affiliations:** a STEM Education Center, Tokai University, 4-1-1 Kitakaname, Hiratuka, Kanagawa 259-1292, Japan

**Keywords:** crystal structure, serine protease inhibitor, treatment of serve acute pancreatitis, anti­coagulant agent

## Abstract

Nafamostat dimesylate {systematic name: [amino({6-[(4-{[amino(iminiumyl)methyl]amino}phenyl)carbonyloxy]naphthalen-2-yl})methylidene]azanium bis(methanesulfonate)}, C_19_H_19_N_5_O_2_^2+^·2CH_3_O_3_S^−^, is a broad-spectrum serine protease inhibitor and has been applied clinically as an anti­coagulant agent during hemodialysis and for treatment of severe acute pancreatitis (SAP). Since nafamostat contains flexible moieties, it is necessary to determine the conformation to understand the structure–activity relationships. The divalent cation has a screw-like motif. The guanidinium group is approximately perpendicular to the naphthyl ring system, subtending a dihedral angle of 84.30 (14)°. In the crystal, the nafamostat mol­ecules form columnar structures surrounded by a hydro­philic region.

## Abstract 2

The crystal structure of nafamostat mesylate is reported, which is a serine protease inhibitor, and has been applied clinically as an anti­coagulant and anti-inflammatory agent.

## Chemical context   

Nafamostat mesylate (I) is the bis­methane­sulfonic salt of 6-amidino-2-naphthyl-4-guanidinobenzoate. It shows broad-spectrum serine protease inhibition effect, and is also a reversible competitive inhibitor as camostat mesylate (II) (Tamura *et al.*, 1977 ▸; Fujii & Hitomi, 1981 ▸; Matsumoto *et al.*, 1989 ▸). Although nafamostat mesylate has been applied clinically with success as an effective anti­coagulant and anti-inflammatory agent during hemodialysis and for treatment of severe acute pancreatitis (Takeda *et al.*, 1989 ▸), the crystal structure has not previously been reported.
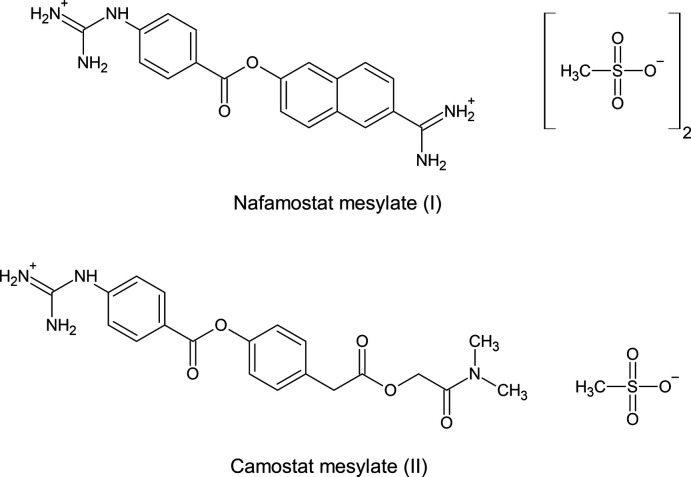


In addition, nafamostat has attracted attention as an inhibitor for the activity of transmembrane protease serine 2 (TMPRSS2), a host cell serine protease that mediates viral cell incursion for influenza virus and coronavirus, thereby inhibiting viral infection and replication (Yamamoto *et al.*, 2016 ▸, 2020 ▸; Hoffmann *et al.*, 2020 ▸). Since nafamostat contains flexible moieties, it is necessary to determine the conformation to understand the structure–activity relationships. The crystal structure of nafamostat mesylate (I) is reported herein. From the crystallographic study, the phenyl­guanidine groups in nafamostat and camostat are essentially similar except for the direction of residual groups.

## Structural commentary   

**Figure 1:**
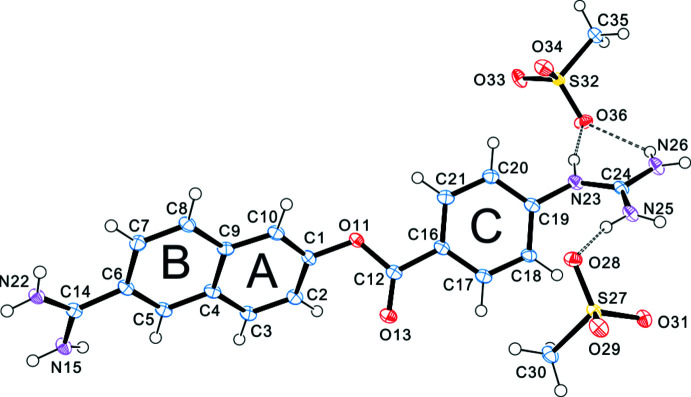
The title compound nafamostat mesylate (I) showing the atom and ring labelling. Displacement ellipsoids are drawn at the 50% probability level.

The nafamostat moiety in the title compound (Fig. 1 ▸) shows a divalent cation with a screw-like motif, which consists of four planar parts: the amidino group, the naphthyl group (rings *A* and *B*), phenyl ring C and the guanidinium group (shown in Fig.1). The dihedral angles between the amidino and naphthyl groups, the naphthyl group and ring *C*, and ring *C* and guanidinium group are 11.35 (13), 44.66 (10) and 51.11 (15)°, respectively. The guanidinium group is approximately perpendicular to the naphthyl group, subtending a dihedral angle of 84.30 (14)°.

The C14—N15 and C14—N22 bond distances [1.319 (3) and 1.311 (3) Å, respectively] indicate a resonance structure in the protonated amidinium group (Table 1 ▸). On the other hand, the bond distances C24—N23 = 1.357 (3), C24—N25 = 1.302 (4) and C24—N26 = 1.325 (3) Å indicate a localized electron on the C24—N25 bond in the protonated guanidinium group.

**Figure 2:**
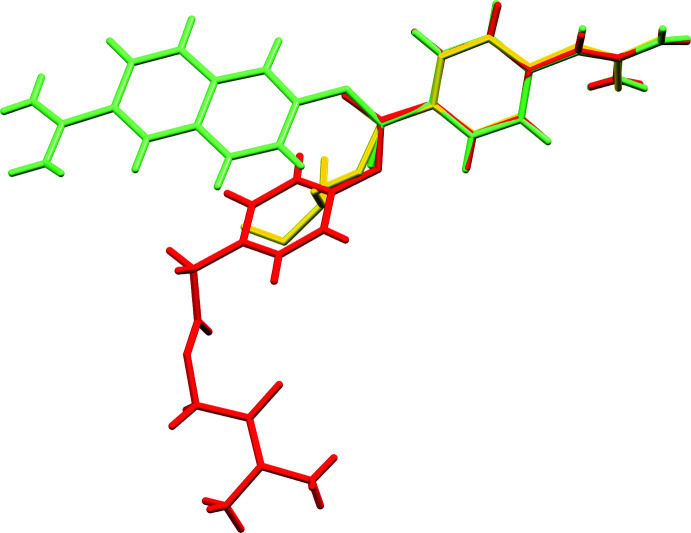
Overlay of the crystal structures of nafamostat moiety (green), camostat moiety (red) and covalently binding partial structure (yellow) of mature nafamostat with Ser441 in the active site from pdb7MEQ (Fraser *et al.*, 2021 ▸), using *Mercury* (Macrae *et al.*, 2020 ▸).

The overlay of nafamostat (green) and camostat (red) is presented in Fig. 2 ▸, in which the r.m.s. deviation is 0.027 Å for phenyl­guanidinium groups. The partial structures are essentially similar, except for the direction of residual groups. Very recently, the crystal structure of human TMPRSS2 in a covalent complex with nafamostat has been solved (Fraser *et al.*, 2021 ▸). The nafamostat in the complex is hydrolysed, and results in phenyl­guanidino acyl­ation of Ser441 (yellow) in the active site. It was considered that the nafamostat moiety may be easily nucleophilic-attacked, approaching from the O13 atom side without steric hindrance.

## Supra­molecular features   

**Figure 3:**
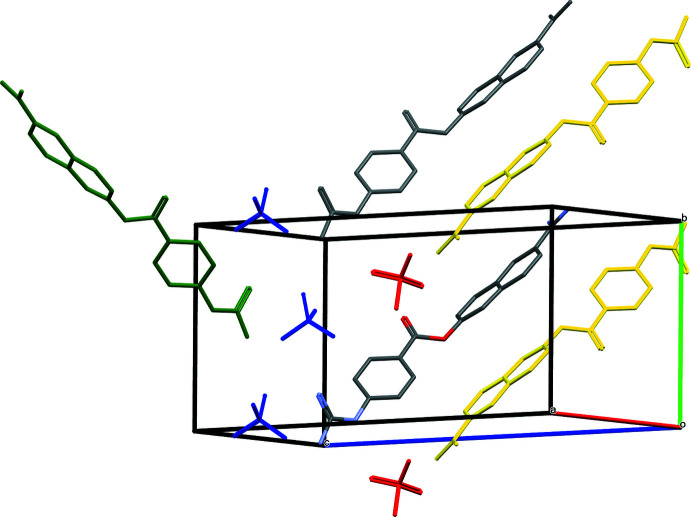
Part of the crystal structure of nafamostat mesylate (I). The naphthyl groups related by the inversion center (yellow) and equivalent (grey) are stacking along the *b*-axis direction, forming a columnar structure. The methane­sulfonate groups containing the S27 and S32 atoms are represented in blue and red, respectively. H atoms have been omitted for clarity.

In the crystal, the naphthyl groups of nafamostat form hydro­phobic columnar structures, shown in Fig. 3 ▸. The naphthyl groups correlated with the inversion center (yellow) are stacking along the *b*-axis direction, in which the perpendicular distances of the centroid of the naphthyl ring system and those at (−*x*, 1 − *y*, 1 − *z*) and (−*x*, 2 − *y*, 1 − *z*) are 3.4208 (8) and 3.5134 (8) Å, respectively.

The columnar structures are surrounded by a hydro­philic region consisting of the methane­sulfonate ions and the guanidinium, imidamidium and ester groups. The two independent methane­sulfonate ions play different roles. The columnar structure inter­calates the methane­sulfonate group (blue) containing the S27 atom, and is linked to three neighbouring guanidinium groups and one di­amine group. Hydrogen bonds [N25—H25*A*⋯O28 = 2.827 (3) and N26—H26*B*⋯O29^vii^ = 2.925 (3) Å; Table 2 ▸] link the mol­ecules, forming an infinite 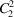
(8) chain, with other hydrogen bonds [N25—H25*B*⋯O29^vi^ = 2.931 (3) and N26—H26*A*⋯O31^vi^ = 2.916 (3) Å] forming an 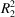
(8) ring.

The columnar structures are also consolidated by the other methane­sulfonate ion (red) containing the S32 atom, which is linked by two opposing amidino groups [N15—H15*A*⋯O33^iii^ =2.854 (3) and N15—H15*B*⋯O34^iv^ = 2.830 (3) Å], related by the inversion center, into an 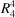
(12) ring. A weak C—H⋯π inter­action is also observed (Table 2 ▸).

## Database survey   

The crystal structures of serine protease inhibitors have been reported for benzamidine (TEKTUY: Barker *et al.*, 1996 ▸), benzamidine HCl (DOHHAJ: Thailambal *et al.*, 1986 ▸) and camostat mesylate (JAMREU: Matsumoto *et al.*, 1989 ▸). Moreover, a search of the Cambridge Structural Database (CSD version 5.42, last updated May 2021; Groom *et al.*, 2016 ▸) yielded another comparable structure, 4-guanidinio­benzoic acid HCl dihydrate (NIQCEW: Light *et al.*, 2007 ▸). Another database search (PDB; Berman *et al.*, 2000 ▸) yielded the crystal structure of human TMPRSS2 in a covalent complex with nafamostat (PDB7MEQ: Fraser *et al.*, 2021 ▸).

## Synthesis and crystallization   

Nafamostat mesylate (CAS No. 82956-11-4) was purchased from Tokyo Chemical Industry Co. Ltd (TCI). A small portion (*ca* 10 mg) was dissolved in a small volume of hot water (*ca* 100 µL), and acetone (*ca* 900 µL) was added slowly until it became cloudy white. On slow cooling to ambient temperature, colourless octa­hedral crystals suitable for X-ray measurements were obtained.

## Refinement   

Crystal data, data collection and structure refinement details at a low temperature (95 K) are summarized in Table 3 ▸. All the H atoms were located in difference-Fourier maps. In the NH or NH_2_ groups, H atoms were freely refined. The C-bound H atoms were included in calculated positions and treated as riding atoms: C—H = 0.95–0.98 Å with *U*_iso_(H) = 1.2–1.5*U*_eq_(C).

## Supplementary Material

Crystal structure: contains datablock(s) I. DOI: 10.1107/S2056989021009245/tx2042sup1.cif

Structure factors: contains datablock(s) I. DOI: 10.1107/S2056989021009245/tx2042Isup2.hkl

Supporting information file. DOI: 10.1107/S2056989021009245/tx2042Isup3.cml

CCDC reference: 2107852

Additional supporting information:  crystallographic information; 3D view; checkCIF report

## Figures and Tables

**Table 1:** Selected bond lengths (Å)

C1—O11	1.391 (3)	C30—S27	1.764 (3)
C12—O13	1.204 (3)	C35—S32	1.762 (3)
C12—O11	1.377 (3)	O28—S27	1.4515 (19)
C14—N22	1.311 (3)	O29—S27	1.4570 (19)
C14—N15	1.319 (3)	O31—S27	1.4700 (18)
C19—N23	1.417 (3)	O33—S32	1.4545 (18)
C24—N26	1.325 (3)	O34—S32	1.4553 (19)
C24—N25	1.302 (4)	O36—S32	1.448 (2)
C24—N23	1.357 (3)		

**Table 2:** Hydrogen-bond geometry (Å, °) *Cg*(*C*) is the center of gravity of phenyl ring *C*.

*D*—H⋯*A*	*D*—H	H⋯*A*	*D*⋯*A*	*D*—H⋯*A*
C18—H18⋯O13^i^	0.95	2.64	3.419 (3)	140
C30—H30*A*⋯O36^ii^	0.98	2.36	3.314 (3)	165
N15—H15*A*⋯O33^iii^	0.93 (4)	1.97 (4)	2.854 (3)	159 (3)
N15—H15*A*⋯O34^iii^	0.93 (4)	2.63 (4)	3.327 (3)	132 (3)
N15—H15*A*⋯S32^iii^	0.93 (4)	2.75 (4)	3.612 (2)	154 (3)
N15—H15*B*⋯O34^iv^	0.85 (4)	2.00 (4)	2.830 (3)	164 (4)
N22—H22*A*⋯O31^v^	0.83 (3)	2.12 (3)	2.928 (3)	162 (3)
N22—H22*B*⋯O33^iii^	0.83 (3)	2.31 (3)	3.018 (3)	144 (3)
N23—H23⋯O36	0.99 (4)	1.88 (5)	2.836 (3)	163 (4)
N23—H23⋯S32	0.99 (4)	2.72 (4)	3.683 (2)	166 (3)
N25—H25*A*⋯O28	0.87 (4)	2.00 (4)	2.827 (3)	159 (3)
N25—H25*A*⋯S27	0.87 (4)	2.86 (4)	3.558 (2)	139 (3)
N25—H25*B*⋯O29^vi^	0.83 (3)	2.12 (3)	2.931 (3)	167 (3)
N26—H26*A*⋯O31^vi^	0.85 (4)	2.10 (4)	2.916 (3)	163 (4)
N26—H26*A*⋯S27^vi^	0.85 (4)	3.01 (4)	3.799 (2)	156 (3)
N26—H26*B*⋯O29^vii^	0.89 (4)	2.46 (4)	2.925 (3)	113 (3)
N26—H26*B*⋯O36	0.89 (4)	2.45 (4)	3.174 (3)	139 (3)
C30—H30*B*⋯*Cg*(C)^vii^	0.98	2.96	3.405 (3)	109

Symmetry codes: (i) -x+1, y-{\script{1\over 2}}, -z+{\script{3\over 2}}; (ii) x, y+1, z; (iii) -x, -y+1, -z+1; (iv) x-1, y+1, z; (v) x-1, -y+{\script{3\over 2}}, z-{\script{1\over 2}}; (vi) -x+2, y-{\script{1\over 2}}, -z+{\script{3\over 2}}; (vii) x, y-1, z.

**Table 3:** Experimental details

Crystal data	
Chemical formula	C_19_H_19_N_5_O_2_^2+^·2CH_3_O_3_S^−^
*M*_r_	539.58
Crystal system, space group	Monoclinic, *P*2_1_/*c*
Temperature (K)	95
*a*, *b*, *c* (Å)	11.0631 (1), 9.7215 (1), 21.9271 (3)
β (°)	96.746 (1)
*V* (Å^3^)	2341.93 (5)
*Z*	4
Radiation type	Cu *K*α
μ (mm^−1^)	2.59
Crystal size (mm)	0.4 × 0.3 × 0.3

Data collection	
Diffractometer	A Rigaku XtaLAB P200
Absorption correction	Multi-scan (*CrysAlis PRO*; Rigaku, 2015 ▸)
*T*_min_, *T*_max_	0.46, 1
No. of measured, independent and observed [*I* > 2σ(*I*)] reflections	4675, 4675, 4457
*R*_int_	0.058
(sin θ/λ)_max_ (Å^−1^)	0.623

Refinement	
*R*[*F*^2^ > 2σ(*F* ^2^)], *wR*(*F* ^2^), *S*	0.055, 0.126, 1.07
No. of reflections	4675
No. of parameters	363
H-atom treatment	H atoms treated by a mixture of independent and constrained refinement
Δρ_max_, Δρ_min_ (e Å^−3^)	0.64, −0.52

Computer programs: *CrysAlis PRO* (Rigaku, 2015 ▸), *SORTAV* (Blessing, 1995 ▸), *SHELXT* (Sheldrick, 2015*a* ▸), *SHELXL* (Sheldrick, 2015*b* ▸), *ORTEP-3 for Windows* and *WinGX* (Farrugia, 2012 ▸), *Mercury* (Macrae *et al.*, 2020 ▸), *PLATON* (Spek, 2020 ▸) and *publCIF* (Westrip, 2010 ▸).

## supplementary crystallographic information

### Crystal data

**Table:**

C_19_H_19_N_5_O_2_^2+^·2CH_3_O_3_S^−^	*F*(000) = 1128
*M**_r_* = 539.58	*D*_x_ = 1.53 Mg m^−^^3^
Monoclinic, *P*2_1_/*c*	Cu *K*α radiation, λ = 1.54184 Å
Hall symbol: -P 2ybc	Cell parameters from 17552 reflections
*a* = 11.0631 (1) Å	θ = 5.0–73.5°
*b* = 9.7215 (1) Å	µ = 2.59 mm^−^^1^
*c* = 21.9271 (3) Å	*T* = 95 K
β = 96.746 (1)°	Octahedron, clear light colourless
*V* = 2341.93 (5) Å^3^	0.4 × 0.3 × 0.3 mm
*Z* = 4	

### Data collection

**Table:**

A Rigaku XtaLAB P200 diffractometer	4675 independent reflections
Radiation source: fine-focus sealed X-ray tube	4457 reflections with *I* > 2σ(*I*)
Graphite monochromator	*R*_int_ = 0.058
φ or ω oscillation scans	θ_max_ = 73.8°, θ_min_ = 4.0°
Absorption correction: multi-scan (CrysAlis PRO; Rigaku, 2015)	*h* = −13→13
*T*_min_ = 0.46, *T*_max_ = 1	*k* = 0→11
4675 measured reflections	*l* = 0→27

### Refinement

**Table:**

Refinement on *F*^2^	Primary atom site location: dual
Least-squares matrix: full	Secondary atom site location: dual
*R*[*F*^2^ > 2σ(*F*^2^)] = 0.055	Hydrogen site location: mixed
*wR*(*F*^2^) = 0.126	H atoms treated by a mixture of independent and constrained refinement
*S* = 1.07	*w* = 1/[σ^2^(*F*_o_^2^) + (0.0324*P*)^2^ + 4.9921*P*] where *P* = (*F*_o_^2^ + 2*F*_c_^2^)/3
4675 reflections	(Δ/σ)_max_ = 0.001
363 parameters	Δρ_max_ = 0.64 e Å^−^^3^
0 restraints	Δρ_min_ = −0.52 e Å^−^^3^
0 constraints	

### Special details

**Table:**

Geometry. All esds (except the esd in the dihedral angle between two l.s. planes) are estimated using the full covariance matrix. The cell esds are taken into account individually in the estimation of esds in distances, angles and torsion angles; correlations between esds in cell parameters are only used when they are defined by crystal symmetry. An approximate (isotropic) treatment of cell esds is used for estimating esds involving l.s. planes.

### Fractional atomic coordinates and isotropic or equivalent isotropic displacement parameters (Å^2^)

**Table:**

	*x*	*y*	*z*	*U*_iso_*/*U*_eq_	
C1	0.1422 (2)	0.5583 (3)	0.56523 (13)	0.0225 (5)	
C2	0.0580 (2)	0.5892 (3)	0.60620 (13)	0.0237 (5)	
H2	0.061778	0.544066	0.644799	0.028*	
C3	−0.0304 (2)	0.6865 (3)	0.58945 (12)	0.0234 (5)	
H3	−0.086637	0.710328	0.617287	0.028*	
C4	−0.0384 (2)	0.7505 (3)	0.53201 (12)	0.0225 (5)	
C5	−0.1241 (2)	0.8550 (3)	0.51578 (12)	0.0229 (5)	
H5	−0.177017	0.883065	0.544597	0.027*	
C6	−0.1333 (2)	0.9184 (3)	0.45831 (13)	0.0229 (5)	
C7	−0.0545 (2)	0.8762 (3)	0.41562 (12)	0.0237 (5)	
H7	−0.060246	0.917718	0.376162	0.028*	
C8	0.0302 (2)	0.7756 (3)	0.43105 (12)	0.0228 (5)	
H8	0.081746	0.747936	0.401556	0.027*	
C9	0.0437 (2)	0.7116 (3)	0.48926 (12)	0.0221 (5)	
C10	0.1352 (2)	0.6151 (3)	0.50758 (12)	0.0224 (5)	
H10	0.191808	0.589251	0.480271	0.027*	
C12	0.3080 (2)	0.4764 (3)	0.63457 (12)	0.0205 (5)	
C14	−0.2214 (2)	1.0326 (3)	0.44424 (12)	0.0203 (5)	
C16	0.4005 (2)	0.3658 (3)	0.64313 (12)	0.0206 (5)	
C17	0.4753 (2)	0.3600 (3)	0.69882 (12)	0.0223 (5)	
H17	0.466534	0.426978	0.729554	0.027*	
C18	0.5632 (2)	0.2563 (3)	0.70998 (12)	0.0222 (5)	
H18	0.61345	0.252049	0.748167	0.027*	
C19	0.5758 (2)	0.1599 (3)	0.66434 (12)	0.0217 (5)	
C20	0.5019 (2)	0.1658 (3)	0.60820 (12)	0.0237 (5)	
H20	0.51147	0.100093	0.577062	0.028*	
C21	0.4145 (2)	0.2678 (3)	0.59814 (12)	0.0229 (5)	
H21	0.363537	0.271047	0.560152	0.027*	
C24	0.7756 (2)	0.0499 (3)	0.69864 (12)	0.0212 (5)	
C30	0.7107 (2)	0.5832 (3)	0.76962 (13)	0.0245 (6)	
H30A	0.661242	0.637008	0.738122	0.037*	
H30B	0.734263	0.641357	0.805571	0.037*	
H30C	0.663287	0.504665	0.781635	0.037*	
C35	0.6293 (2)	−0.4167 (3)	0.57039 (13)	0.0262 (6)	
H35A	0.717501	−0.403331	0.578279	0.039*	
H35B	0.607172	−0.441466	0.527188	0.039*	
H35C	0.604676	−0.490755	0.596665	0.039*	
N15	−0.3033 (2)	1.0565 (3)	0.48204 (11)	0.0238 (5)	
N22	−0.2175 (2)	1.1128 (2)	0.39642 (11)	0.0214 (5)	
N23	0.65733 (19)	0.0475 (2)	0.67377 (11)	0.0242 (5)	
N25	0.8365 (2)	0.1631 (2)	0.71094 (12)	0.0242 (5)	
N26	0.8283 (2)	−0.0718 (2)	0.70825 (12)	0.0252 (5)	
O11	0.23387 (15)	0.46189 (19)	0.57993 (8)	0.0217 (4)	
O13	0.29656 (16)	0.5684 (2)	0.67019 (9)	0.0255 (4)	
O28	0.80155 (16)	0.44602 (19)	0.68474 (8)	0.0234 (4)	
O29	0.91327 (16)	0.64512 (19)	0.72854 (9)	0.0252 (4)	
O31	0.90733 (15)	0.43603 (19)	0.78786 (8)	0.0221 (4)	
O33	0.42506 (16)	−0.2921 (2)	0.57331 (10)	0.0308 (5)	
O34	0.59193 (17)	−0.1572 (2)	0.54595 (9)	0.0282 (4)	
O36	0.59191 (19)	−0.2314 (2)	0.65057 (9)	0.0308 (4)	
S27	0.84252 (5)	0.52334 (6)	0.73995 (3)	0.01897 (15)	
S32	0.55448 (5)	−0.26334 (6)	0.58663 (3)	0.01882 (15)	
H22A	−0.168 (3)	1.102 (3)	0.3710 (14)	0.017 (7)*	
H25B	0.902 (3)	0.162 (3)	0.7333 (14)	0.019 (7)*	
H22B	−0.266 (3)	1.177 (3)	0.3897 (13)	0.019 (7)*	
H25A	0.806 (3)	0.244 (4)	0.7016 (17)	0.039 (10)*	
H26B	0.787 (3)	−0.150 (4)	0.7025 (17)	0.045 (10)*	
H15A	−0.354 (3)	1.132 (4)	0.4725 (17)	0.043 (10)*	
H26A	0.905 (4)	−0.078 (4)	0.7167 (18)	0.051 (11)*	
H15B	−0.323 (3)	0.994 (4)	0.5063 (18)	0.043 (10)*	
H23	0.627 (4)	−0.042 (5)	0.6577 (19)	0.062 (12)*	

### Atomic displacement parameters (Å^2^)

**Table:**

	*U*^11^	*U*^22^	*U*^33^	*U*^12^	*U*^13^	*U*^23^
C1	0.0136 (11)	0.0182 (13)	0.0341 (14)	0.0017 (9)	−0.0030 (10)	−0.0012 (10)
C2	0.0163 (12)	0.0241 (14)	0.0307 (14)	−0.0010 (10)	0.0023 (10)	0.0018 (11)
C3	0.0134 (11)	0.0253 (14)	0.0324 (14)	0.0000 (10)	0.0060 (10)	−0.0028 (11)
C4	0.0129 (11)	0.0224 (13)	0.0319 (14)	−0.0016 (10)	0.0021 (10)	−0.0035 (11)
C5	0.0141 (11)	0.0245 (14)	0.0306 (13)	−0.0018 (10)	0.0047 (10)	−0.0030 (11)
C6	0.0126 (11)	0.0194 (13)	0.0359 (14)	−0.0009 (9)	0.0002 (10)	−0.0011 (11)
C7	0.0184 (12)	0.0250 (14)	0.0271 (13)	−0.0014 (10)	0.0005 (10)	0.0011 (10)
C8	0.0160 (12)	0.0217 (13)	0.0301 (13)	0.0023 (10)	0.0008 (10)	−0.0031 (10)
C9	0.0156 (11)	0.0249 (14)	0.0256 (13)	−0.0039 (10)	0.0015 (10)	−0.0012 (10)
C10	0.0177 (12)	0.0224 (14)	0.0277 (13)	−0.0004 (10)	0.0051 (10)	−0.0012 (10)
C12	0.0138 (11)	0.0196 (13)	0.0286 (13)	−0.0014 (9)	0.0050 (10)	−0.0004 (10)
C14	0.0114 (11)	0.0187 (13)	0.0303 (13)	−0.0028 (9)	0.0000 (9)	−0.0012 (10)
C16	0.0126 (11)	0.0201 (13)	0.0293 (13)	−0.0013 (9)	0.0033 (9)	0.0019 (10)
C17	0.0170 (12)	0.0255 (14)	0.0244 (12)	−0.0037 (10)	0.0018 (10)	0.0002 (10)
C18	0.0182 (12)	0.0219 (13)	0.0259 (13)	−0.0015 (10)	0.0000 (10)	−0.0001 (10)
C19	0.0144 (11)	0.0218 (13)	0.0290 (13)	−0.0016 (10)	0.0029 (10)	0.0020 (10)
C20	0.0166 (12)	0.0250 (14)	0.0290 (13)	0.0003 (10)	0.0010 (10)	0.0008 (11)
C21	0.0209 (12)	0.0217 (13)	0.0270 (13)	−0.0021 (10)	0.0069 (10)	0.0000 (10)
C24	0.0142 (11)	0.0256 (14)	0.0247 (12)	0.0026 (10)	0.0057 (9)	0.0020 (10)
C30	0.0151 (12)	0.0286 (15)	0.0301 (14)	0.0051 (10)	0.0035 (10)	0.0020 (11)
C35	0.0194 (12)	0.0225 (14)	0.0359 (15)	0.0023 (10)	0.0004 (11)	0.0014 (11)
N15	0.0169 (10)	0.0226 (12)	0.0324 (12)	0.0037 (9)	0.0054 (9)	0.0062 (10)
N22	0.0149 (10)	0.0217 (12)	0.0280 (12)	0.0019 (9)	0.0037 (9)	0.0004 (9)
N23	0.0137 (10)	0.0245 (12)	0.0334 (12)	0.0027 (9)	−0.0012 (9)	−0.0001 (10)
N25	0.0160 (11)	0.0187 (12)	0.0378 (13)	0.0028 (9)	0.0037 (10)	0.0035 (10)
N26	0.0146 (11)	0.0202 (12)	0.0409 (14)	0.0014 (9)	0.0034 (9)	0.0005 (10)
O11	0.0142 (8)	0.0238 (10)	0.0267 (9)	0.0021 (7)	0.0002 (7)	−0.0028 (7)
O13	0.0184 (9)	0.0270 (10)	0.0310 (10)	0.0029 (7)	0.0019 (7)	−0.0023 (8)
O28	0.0234 (9)	0.0202 (9)	0.0264 (9)	0.0013 (7)	0.0021 (7)	−0.0010 (7)
O29	0.0202 (9)	0.0200 (10)	0.0358 (10)	−0.0005 (7)	0.0059 (8)	0.0036 (8)
O31	0.0136 (8)	0.0236 (10)	0.0288 (9)	0.0045 (7)	0.0012 (7)	0.0060 (7)
O33	0.0126 (9)	0.0248 (10)	0.0547 (13)	0.0006 (7)	0.0027 (8)	0.0066 (9)
O34	0.0288 (10)	0.0214 (10)	0.0358 (11)	0.0005 (8)	0.0102 (8)	0.0067 (8)
O36	0.0398 (11)	0.0225 (10)	0.0282 (10)	0.0011 (8)	−0.0038 (8)	0.0013 (8)
S27	0.0124 (3)	0.0184 (3)	0.0264 (3)	0.0017 (2)	0.0034 (2)	0.0019 (2)
S32	0.0117 (3)	0.0167 (3)	0.0281 (3)	0.0007 (2)	0.0021 (2)	0.0025 (2)

### Geometric parameters (Å, º)

**Table:**

C1—O11	1.391 (3)	C35—S32	1.762 (3)
C1—C2	1.401 (4)	C35—H35C	0.98
C1—C10	1.373 (4)	C35—H35B	0.98
C10—H10	0.95	C35—H35A	0.98
C12—O13	1.204 (3)	C4—C9	1.430 (4)
C12—O11	1.377 (3)	C4—C5	1.407 (4)
C12—C16	1.481 (3)	C5—H5	0.95
C14—N22	1.311 (3)	C5—C6	1.396 (4)
C14—N15	1.319 (3)	C6—C7	1.413 (4)
C16—C21	1.393 (4)	C6—C14	1.486 (3)
C16—C17	1.393 (4)	C7—H7	0.95
C17—H17	0.95	C7—C8	1.369 (4)
C17—C18	1.402 (4)	C8—H8	0.95
C18—H18	0.95	C8—C9	1.412 (4)
C18—C19	1.390 (4)	C9—C10	1.403 (4)
C19—N23	1.417 (3)	N15—H15B	0.85 (4)
C19—C20	1.397 (4)	N15—H15A	0.93 (4)
C2—H2	0.95	N22—H22B	0.83 (3)
C2—C3	1.379 (4)	N22—H22A	0.83 (3)
C20—H20	0.95	N23—H23	0.99 (4)
C20—C21	1.384 (4)	N25—H25B	0.83 (3)
C21—H21	0.95	N25—H25A	0.87 (4)
C24—N26	1.325 (3)	N26—H26B	0.89 (4)
C24—N25	1.302 (4)	N26—H26A	0.85 (4)
C24—N23	1.357 (3)	O28—S27	1.4515 (19)
C3—H3	0.95	O29—S27	1.4570 (19)
C3—C4	1.398 (4)	O31—S27	1.4700 (18)
C30—S27	1.764 (3)	O33—S32	1.4545 (18)
C30—H30C	0.98	O34—S32	1.4553 (19)
C30—H30B	0.98	O36—S32	1.448 (2)
C30—H30A	0.98		
			
C10—C1—O11	116.5 (2)	C21—C20—H20	120.2
C10—C1—C2	122.3 (2)	C19—C20—H20	120.2
O11—C1—C2	121.2 (2)	C20—C21—C16	120.7 (3)
C3—C2—C1	118.7 (2)	C20—C21—H21	119.6
C3—C2—H2	120.6	C16—C21—H21	119.6
C1—C2—H2	120.6	N25—C24—N26	121.0 (2)
C2—C3—C4	120.8 (2)	N25—C24—N23	123.3 (2)
C2—C3—H3	119.6	N26—C24—N23	115.7 (2)
C4—C3—H3	119.6	S27—C30—H30A	109.5
C3—C4—C5	121.2 (2)	S27—C30—H30B	109.5
C3—C4—C9	119.7 (2)	H30A—C30—H30B	109.5
C5—C4—C9	119.1 (2)	S27—C30—H30C	109.5
C6—C5—C4	121.5 (2)	H30A—C30—H30C	109.5
C6—C5—H5	119.3	H30B—C30—H30C	109.5
C4—C5—H5	119.3	S32—C35—H35A	109.5
C5—C6—C7	119.0 (2)	S32—C35—H35B	109.5
C5—C6—C14	119.6 (2)	H35A—C35—H35B	109.5
C7—C6—C14	121.3 (2)	S32—C35—H35C	109.5
C8—C7—C6	120.1 (2)	H35A—C35—H35C	109.5
C8—C7—H7	120	H35B—C35—H35C	109.5
C6—C7—H7	120	C14—N15—H15A	116 (2)
C7—C8—C9	122.3 (2)	C14—N15—H15B	120 (3)
C7—C8—H8	118.8	H15A—N15—H15B	121 (3)
C9—C8—H8	118.8	C14—N22—H22A	123 (2)
C10—C9—C8	123.3 (2)	C14—N22—H22B	121 (2)
C10—C9—C4	118.7 (2)	H22A—N22—H22B	116 (3)
C8—C9—C4	117.9 (2)	C24—N23—C19	127.7 (2)
C1—C10—C9	119.6 (2)	C24—N23—H23	115 (2)
C1—C10—H10	120.2	C19—N23—H23	117 (2)
C9—C10—H10	120.2	C24—N25—H25B	121 (2)
O13—C12—O11	122.9 (2)	C24—N25—H25A	122 (2)
O13—C12—C16	125.5 (2)	H25B—N25—H25A	116 (3)
O11—C12—C16	111.6 (2)	C24—N26—H26B	122 (2)
N22—C14—N15	119.1 (2)	C24—N26—H26A	120 (3)
N22—C14—C6	121.9 (2)	H26B—N26—H26A	117 (4)
N15—C14—C6	119.0 (2)	C12—O11—C1	118.4 (2)
C17—C16—C21	119.3 (2)	O28—S27—O29	113.51 (11)
C17—C16—C12	118.1 (2)	O28—S27—O31	112.04 (11)
C21—C16—C12	122.6 (2)	O29—S27—O31	111.41 (11)
C16—C17—C18	120.7 (2)	O28—S27—C30	106.72 (12)
C16—C17—H17	119.7	O29—S27—C30	106.21 (12)
C18—C17—H17	119.7	O31—S27—C30	106.39 (12)
C19—C18—C17	119.0 (2)	O36—S32—O33	113.48 (13)
C19—C18—H18	120.5	O36—S32—O34	111.82 (12)
C17—C18—H18	120.5	O33—S32—O34	110.96 (12)
C18—C19—C20	120.6 (2)	O36—S32—C35	106.84 (13)
C18—C19—N23	122.1 (2)	O33—S32—C35	105.73 (12)
C20—C19—N23	117.2 (2)	O34—S32—C35	107.57 (12)
C21—C20—C19	119.7 (3)		

### Hydrogen-bond geometry (Å, º)

*Cg*(*C*) is the center of gravity of phenyl ring *C*.

**Table:**

*D*—H···*A*	*D*—H	H···*A*	*D*···*A*	*D*—H···*A*
C18—H18···O13^i^	0.95	2.64	3.419 (3)	140
C30—H30*A*···O36^ii^	0.98	2.36	3.314 (3)	165
N15—H15*A*···O33^iii^	0.93 (4)	1.97 (4)	2.854 (3)	159 (3)
N15—H15*A*···O34^iii^	0.93 (4)	2.63 (4)	3.327 (3)	132 (3)
N15—H15*A*···S32^iii^	0.93 (4)	2.75 (4)	3.612 (2)	154 (3)
N15—H15*B*···O34^iv^	0.85 (4)	2.00 (4)	2.830 (3)	164 (4)
N22—H22*A*···O31^v^	0.83 (3)	2.12 (3)	2.928 (3)	162 (3)
N22—H22*B*···O33^iii^	0.83 (3)	2.31 (3)	3.018 (3)	144 (3)
N23—H23···O36	0.99 (4)	1.88 (5)	2.836 (3)	163 (4)
N23—H23···S32	0.99 (4)	2.72 (4)	3.683 (2)	166 (3)
N25—H25*A*···O28	0.87 (4)	2.00 (4)	2.827 (3)	159 (3)
N25—H25*A*···S27	0.87 (4)	2.86 (4)	3.558 (2)	139 (3)
N25—H25*B*···O29^vi^	0.83 (3)	2.12 (3)	2.931 (3)	167 (3)
N26—H26*A*···O31^vi^	0.85 (4)	2.10 (4)	2.916 (3)	163 (4)
N26—H26*A*···S27^vi^	0.85 (4)	3.01 (4)	3.799 (2)	156 (3)
N26—H26*B*···O29^vii^	0.89 (4)	2.46 (4)	2.925 (3)	113 (3)
N26—H26*B*···O36	0.89 (4)	2.45 (4)	3.174 (3)	139 (3)
C30—H30*B*···*Cg*(C)^vii^	0.98	2.96	3.405 (3)	109

Symmetry codes: (i) −*x*+1, *y*−1/2, −*z*+3/2; (ii) *x*, *y*+1, *z*; (iii) −*x*, −*y*+1, −*z*+1; (iv) *x*−1, *y*+1, *z*; (v) *x*−1, −*y*+3/2, *z*−1/2; (vi) −*x*+2, *y*−1/2, −*z*+3/2; (vii) *x*, *y*−1, *z*.
